# Protein Phosphorylation in Cancer: Role of Nitric Oxide Signaling Pathway

**DOI:** 10.3390/biom11071009

**Published:** 2021-07-10

**Authors:** Xinran Liu, Yiping Zhang, Yijie Wang, Meiwen Yang, Fenfang Hong, Shulong Yang

**Affiliations:** 1 Department of Physiology, College of Medicine, Nanchang University, Nanchang 330006, China; liuxinran_sheila@163.com (X.L.); yipingzhang53@yeah.net (Y.Z.); yijiewyj0406@163.com (Y.W.); 2 Queen Mary School, Nanchang University, Nanchang 330006, China; 3 Department of Physiology, Fuzhou Medical College, Fuzhou 344000, China; y18379441123@126.com; 4 Department of Nurse, Nanchang University Hospital, Nanchang 330006, China; 5 Experimental Center of Pathogen Biology, Nanchang University, Nanchang 330031, China

**Keywords:** nitric oxide, cancer, protein phosphorylation, signaling pathways

## Abstract

Nitric oxide (NO), a free radical, plays a critical role in a wide range of physiological and pathological processes. Due to its pleiotropic function, it has been widely investigated in various types of cancers and is strongly associated with cancer development. Mounting pieces of evidence show that NO regulates various cancer-related events, which mainly depends on phosphorylating the key proteins in several signaling pathways. However, phosphorylation of proteins modulated by NO signaling pathway may lead to different effects in different types of cancer, which is complex and remains unclear. Therefore, in this review, we focus on the effect of protein phosphorylation modulated by NO signaling pathway in different types of cancers including breast cancer, lung cancer, prostate cancer, colon cancer, gastric cancer, pancreatic cancer, ovarian cancer, and neuroblastoma. Phosphorylation of key proteins, including p38 MAPK, ERK, PI3K, STAT3, and p53, modified by NO in various signaling pathways affects different cancer-related processes including cell apoptosis, proliferation, angiogenesis, metastasis, and several cancer therapies. Our review links the NO signaling pathway to protein phosphorylation in cancer development and provides new insight into potential targets and cancer therapy.

## 1. Introduction

The development of the tumor is influenced by multiple factors including heredity and environment. In the genetic central dogma, genetic information is transmitted from DNA to mRNA, which is called transcription. Then, RNA is translated into protein, which will process by post-translational modifications (PTM) later on. Studies have shown that mRNA abundance in tissues is not completely consistent with protein abundance [1]. Therefore, the gene does not account for all the phenotypes. PTM is the process of covalent processing of a protein after translation by adding chemical groups to one or more amino acid residues, which can change the physicochemical properties of the protein, thereby influencing the spatial conformation and activity state of the protein, subcellular localization and protein interactions. More than 300 types of PTM are known, but only few have been studied, including phosphorylation, acetylation, ubiquitination, N-glycosylation, nitrification, propionylation, etc. [2].

Among them, protein phosphorylation is the most common and important in PTM. About 30% of the human proteome is phosphorylated, which is involved in almost all life processes of the cell, such as cell division, protein decomposition, signal transduction, gene expression regulation, and protein interaction [3]. However, when there are mutations at the site of protein phosphorylation, it may lead to the occurrence and development of a malignant tumor. Since the enzymes are proteins, the abnormal enzymes may lead to abnormal cell life activities and further lead to carcinogenesis [4]. Many phosphorylation pathways including MAPK, PI3K/Akt, tyrosine kinase, Cadherin–catenin complex, Cyclin-dependent kinase, NF-kappaB and IkappaB proteins, TGF-β Signaling, etc. play an important role in cancer development [5]. 

Specifically, protein phosphorylation promotes the occurrence and development of cancer in the following aspects: inducing the cancer cell proliferation, inhibiting the cancer cell apoptosis, inducing cancer cells invasion and metastasis, inducing cancer cells angiogenesis and inducing cancer stem cells proliferation. Therefore, an in-depth understanding of the relevant mechanisms and signal pathways of protein phosphorylation disorders and related regulatory enzymes can help us to further understand the pathogenesis of cancer and screen tumor-related markers and target molecules, which is also of great significance to the research for potential targeted therapeutic antitumor drugs.

Nitric oxide (NO) is mainly oxidized from L-arginine by the catalyzation of nitric oxide synthase (NOS). As an intracellular signal, it functions in the cardiovascular, neural and immune systems. Depending on different NOS isoforms, NO exhibits different characteristics and functions. Neural NOS (nNOS) and endothelial NOS (eNOS) produce small amounts of NO, which can act as a neurotransmitter, vasodilator, or assist in the release of insulin. While inducible NOS (iNOS) -induces large amounts of NO to fight against pathogens [6]. The effect of NO on cancer cells is dichotomous, which may depend on the type of cancer and the isoforms of NOS [7]. It is commonly believed that iNOS-induced NO promotes carcinogenesis and is associated with lower survival and chemoresistance [8]. However, this idea is still under debate until now. Some studies have also shown that iNOS and iNOS-induced NO act as cancer inhibitors in some cases [9]. 

Furthermore, the concentration and duration of NO exposure also influence its functions. Usually, NO regulates physiological pathways through the soluble-guanylyl-cyclase (sGC)–cGMP pathway and S-nitrosylation [9]. At a low concentration, along with NO derivatives such as nitrite and nitrate, NO may promote the chemoresistance and metastasis of cancer [10]. NO induces nitro-oxidative stress and interferes with the balance of redox, resulting in carcinogenesis [11]. In addition, NO production by cancer cells plays a role in activating signaling pathways involved in proliferation, angiogenesis, and metabolism [11,12]. While at a high concentration, accompanied by NO derivatives such as nitrite and nitrate, NO is more prone to upregulate cancer cell apoptosis and has a cytotoxic function [10,13].

**Figure 1:**
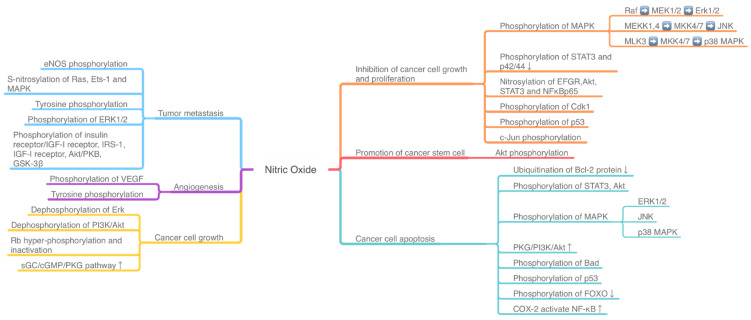
The NO-induced phosphorylation pathway influences the tumor development and death. ERK: extracellular signal-regulated kinase; PI3K: phosphoinositide 3-kinase; MAPK: mitogen-activated protein kinases; MEK: MAPK kinase; MKK: MAPK kinase kinase; ETS-1: E26 avian erythroblastosis virus transcription factor-1; VEGF: vascular endothelial-derived growth factor; JNK: Jun NH2-terminal kinase; Rb: retinoblastoma protein; FOXO: Forkhead box-O; IGF: insulin/insulin-like growth factor; GSK-3β: glycogen synthase kinase-3β.

The effect of NO-related pathway on cancer is the result of many pathways being intertwined, coordinated and even antagonistic. The activity of mitogen-activated protein kinases (MAPKs), phosphoinositide 3-kinase (PI3K)/Akt, TNFα/NF-κB, IL6/STAT3, and other pathways, along with many pathway-related proteins, may induce a dual effect of NO on cancer cells (Figure 1). The activation or inhibition of these signaling pathways is correlated with the phosphorylation and dephosphorylation of related proteins and enzymes in cancer cells (Figure 1, Table A1 Appendix A).

Mitogen-activated protein kinases (MAPKs) are a family of kinases that transmit the signal from the cell membrane to the nucleus, involved in controlling cell proliferation, differentiation, death, and even malignancies and pathogenesis of tumor. There are three major signaling pathways in MAPK cascades. Firstly, the extracellular signal-regulated kinase (ERK)-1/2 targets both membrane proteins and transcription factors, which may function in cancer cell proliferation and survival. The second pathway is cJun NH_2_-terminal kinases (JNK), which play a role in cell apoptosis and oncogenesis. The third one is the p38 MAPK pathway, with its double roles in cancer including inducing and inhibiting leukemia cell proliferation [14].

PI3K/Akt/mTOR signaling pathway mutation is common in various cancer types. PI3K is one of the phosphatidylinositol kinases. When binding with growth factor receptors such as EGFR, PI3K can induce the conformational change of the Akt protein and activate or inhibit downstream substrate via phosphorylation, thus regulating cell proliferation, differentiation, apoptosis, and migration. Akt can also activate IKK and has crosstalk with NF-κB. mTOR is the downstream target of PI3K/Akt, whose downstream transcriptional factors include HIF1α, c-Myc, and FoxO [15].

## 2. Protein Phosphorylation in Different Cancer Induced by NO

In this section, we review the effect of NO signaling pathway to protein phosphorylation in different types of cancers including breast cancer, lung cancer, prostate cancer, colon cancer, gastric cancer, pancreatic cancer, ovarian cancer, and neuroblastoma.

### 2.1. Breast Cancer

**Figure 2:**
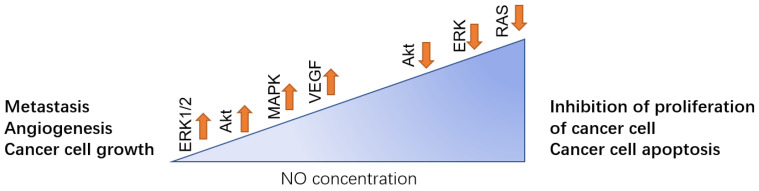
In breast cancer, different NO concentration levels play the opposite role. Low level of NO can facilitate the metastasis to other site of body such as lung, angiogenesis of the tumor and promote the cancer cell growth, which is mainly regulated by activation of ERK1/2, Akt, MAPK, and VEGF. While in high concentration, NO can downregulate those pathways, thus inhibiting the proliferation of the cancer cell and inducing cell apoptosis.

With its increasing morbidity, breast cancer has become one of the most common malignant tumors in women, and also the seventh most common cancer in China. Many studies have indicated that there is a correlation between NO expression and breast cancer [16,17]. Different concentrations of NO have the functions of both inducing and inhibiting cancer development depending on the phosphorylation of NO downstream and upstream regulators. Generally, a low concentration of NO promotes the development of breast cancer by inducing cancer cell growth, metastasis, Epithelial-Mesenchymal Transition (EMT), angiogenesis, invasion, and migration. While a higher concentration of NO inhibits cancer cell growth and facilitates cancer cell apoptosis (Figure 2).

Sen S et al., found that tumor cell growth and proliferation are induced by mitochondrial-associated NOS, which keeps Akt and ERK1/2 in a phosphorylated state [18]. Moreover, Prueitt et al., found that phosphorylation of Akt has a strong correlation with NOS2, which is a synthase of NO [19]. Later, Ridnour LA et al., conducted a further study, which suggested that NO induces the phosphorylation of Akt in human breast cancer cell [20]. S-nitrosylation is one of the PTMs that is mediated by NO. Covalent NO is bound to the thiol side chain of a cysteine residue. It is reported that a high level of NO leads to activation of S-nitrosylation in breast cancer. S-nitrosylation of Ras contributes to the activation of Ets-1, which depends on the phosphorylation of MAPK and causing the metastasis of cancer [21]. Moreover, low production of NO was related to a decreased phosphorylation of eNOS, which is an indicator of the metastasis of breast cancer to other sites of the body at the early stage [19]. When cancer cells reach the site of metastasis, they experience MET (mesenchymal epithelial transition). In these cells, overconcentration of NO is prevalent [22]. It is reported that in the murine breast cancer cell line, glucocorticoids can induce angiogenesis through vascular endothelial-derived growth factor (VEGF) by increasing NO signaling. Also, it indicated that DNA damage and repair may be related to the phosphorylation of γ-H2AX foci and RAD51 foci, which is induced by NO [23]. However, a high concentration of NO inhibits the growth of breast cancer. Human breast cancer cell apoptosis is induced by NO-induced MKP-1 followed by dephosphorylation of ERK, which then leads to dephosphorylation of Akt [24]. Moreover, the upregulation of the Raf/MEK/ERK and PI-3 kinase/Akt pathways of NO are mediated by RAS. Inactivation of RAS can weaken the function of NO [25].

### 2.2. Lung Cancer

With the worsening air pollution and an increasing number of smokers, the morbidity and mortality of lung cancer have ranked first place in all kinds of cancer. At the same time, lung is also a main metastasis site for other tumors such as colon cancer and breast cancer [26]. Many studies have shown that the phosphorylation of NO downstream signal pathway is correlated to the lung cancer cell proliferation, metastasis, angiogenesis, and cancer stem cell. Pamela L. Rice et al., found that NO promotes MEK1/2 phosphorylation, which induces tumor proliferation and growth in lung cancer [27]. Also, NOS overproduced NO can lead to tyrosine phosphorylation and p53 gene accumulation in the human liver epithelial cells [28]. iNOS induced NO can promote angiogenesis in lung cancer. In iNOS knockout mice, vascular endothelial growth factor decreased [29]. Furthermore, NOS-induced NO may exert its effects by influencing tyrosine phosphorylation of proteins and matrix metalloproteinase (MMP) expression in the sprouting tips of nascent capillaries [30]. The cancer stem cell is an important factor in the occurrence and development of malignant tumors. NO can increase cancer stem cell and stemness related protein in human lung cancer. NO promotes Akt-dependent phosphorylation of Caveolin-1 at tyrosine 14 and disrupts the Cav-1: Oct4 complex, then promotes the CSC-regulatory activity [31]. iNOS is a downstream mediator of activated Src kinase in lung cancer, which is an important factor of cell proliferation and metastasis in cancer. The study showed that iNOS is phosphorylated on a tyrosine residue by Src kinase in human alveolar type II epithelium-like lung carcinoma cell line. Therefore, inhibition of Src can be a promising therapy at the late stage of lung cancer [32]. Moreover, NO can increase cell death resistance in the cisplatin-treated lung cancer cell. A study showed that NO can nitrosylate the Bcl-2 protein and inhibit its ubiquitination, which reverses the anti-tumor function of cisplatin [33].

### 2.3. Prostate Cancer

In the study of prostate cancer to chemotherapy under hypoxia, PC-3 prostate cancer cells were treated with NO-sulindac under hypoxic conditions in vitro. A reduced ability of tumor cells to adapt to hypoxia and suppressed expression of hypoxia-inducible factor-1α (HIF-1α) was observed under NO-sulindac treatment [34]. HIF-1α is a transcription factor that regulates oxygen homeostasis and is thought to be related with tumor progression, angiogenesis, metastasis, and resistance [35], which may be responsible for chemoresistance under hypoxia [34]. The NO-sulindac can inhibit invasion and increase survival rate through suppressing the activity of hypoxia response element (HRE) promoter and inhibiting HIF-1α translocation via inhibiting Akt phosphorylation under hypoxia [34]. Thus, it can be a therapeutic agent for the treatment of prostate cancer in hypoxic conditions. However, in addition to the suppression of cancer, NO may also have the opposite effect. It had been mentioned by Baltaci S et al., that iNOS-induced NO played a facilitating role in the progression of prostatic tumorigenesis. Compared to the benign prostate tumor, iNOS expression was higher in prostatic carcinomas, and this could also be used for immunohistochemistry or biological function study of prostate cancers [36].

### 2.4. Colon Cancer

NO can suppress colon cancer cell growth by inhibiting the cell cycle. NO-aspirin (NO-ASA) as a more potential anticancer agent than aspirin, can modulate the phase transition proteins to arrest the tumor cell cycle at the G2/M phase in colon, pancreas, and breast cancer.

It increases Cdk1 phosphorylation while preventing dephosphorylation of Thr14 and Thr15 residues of Cdk1 by reducing the level of cdc25 [37]. Furthermore, NO-ASA induced-oxidative stress disturbs cyclin D at Cys285, which plays a role in the phosphorylation of Thr286 and/or Thr288, resulting in degradation of cyclin D. The arrestment of cell cycle is also enhanced by the upregulating of reactive oxygen species (ROS) production, which can interfere with the cell cycle through oxidizing/reducing cysteine residues on regulatory factors [37,38].

Besides, NO-ASA also interacts with MAPK activation to inhibit colon cancer cell growth. By regulating the MAPK cascades, it stimulates the phosphorylation of p38 MAP kinases and JNK, which further phosphorylate the cJun and activate transcription factor 2 (ATF-2). The effects of NO-ASA at low concentrations on the phosphorylation of ERK-1/2 and Akt are not significant. However, in the presence of high concentrations of NO-ASA (100µM), phosphorylated ERK2 is significantly increased, while phosphorylation of Akt was shown to be inhibited for a certain period [14].

In addition to the above-mentioned effects, NO can inhibit the cell cycle and cause apoptosis in colon cancer cells by phosphorylating serine residues of p53 [39,40]. Activation of p53 can upregulate p21 expression and ultimately lead to cell cycle arrest and cell apoptosis [40]. The overexpression of iNOS enhances the activation of p53 by radiation therapy, which also demonstrates that NO acts as an apoptotic agent in cancer cells through the phosphorylation of p53 [40].

Actually, NO is prone to see dual effects in many pathophysiological activities. Its biological functions are related to the microenvironment concentration, cell susceptibility, and nitric oxide synthase isoforms. Increased levels of NOS expression have been seen to accompany the development of colon and pancreatic cancers. The auguring NO can interact with oncoproteins and signaling pathways that related to tumor progression [41].

Retinoblastoma protein (Rb) is inactivated by iNOS-induced NO in colitis. With a low concentration, NO directs multiple pathways including sGC/cGMP pathway, PI3K/AKT and MAPK pathway to synergistically engage in Rb hyperphosphorylation and prevent pRb-E2F1 pathway-induced apoptosis [42]. Although there is no clear point to the relationship between pRB hyperphosphorylation and colon cancer, it can be speculated that its role in colon cancer development may through a chronic inflammation-related carcinogenesis mechanism.

Furthermore, iNOS-induced NO is confirmed to play an important role in the metastasis, invasion and angiogenesis of colon cancer. NO-induced phosphorylation of ERK-1/2 peaks after 2h in colon adenocarcinoma cell WiDr. Functioning through the NO-cGMP-PKG-ERK1/2 pathway and nuclear translocation of AP-1, NO can upregulate MMP-2/9 expression and induce translocation of Fos-related antigen-1 (Fra-1) and Fos-related antigen-2 (Fra-2) [43]. MMP induce proteolysis and disrupts intercellular connections, therefore the upregulation of MMP can lead to metastasis and invasion of cancer cells.

### 2.5. Gastric Cancer

In gastric cancer cells, NO mainly impacts cell proliferation through regulating protein phosphorylation in several signaling pathways. Evidence obtained from the BGC-823 gastric cancer cell line shows that exposure to SNP at various concentration for 24 h inhibits the cell proliferation and Akt phosphorylation, suggesting that Akt may participate in NO-induced anti-proliferation in gastric cancer [44]. Moreover, type II cGMP-dependent protein kinase (PKG II) may also play an essential role in regulating cell proliferation. Xiaoyuan Y et al., increased the expression of PKG II by infecting AGS gastric cancer cell line with an adenoviral construct encoding PKG II cDNA (Ad-PKG II) and observed the inhibition of AGS cell proliferation induced by SNP. In addition, they also observed the significant inhibition of EGFR and ERK phosphorylation only in AGS cells that express a high level of PKG II [45]. Based on these results, it is indicated that SNP may inhibit the gastric cancer cell proliferation through activating PKG II to inhibit the EGF-induced EGFR phosphorylation, which in turn decreases the phosphorylation of ERK.

### 2.6. Pancreatic Cancer

Similar to colon cancer, NO has a dual function in the development of pancreatic cancer. NO leads to protective or anti-cancer cells effects exhibiting a profile associated with iNOS expression, which mainly induces a large amount of NO to respond to the stimulations.

In a study of pancreatic ductal adenocarcinoma (PDAC), high expression of iNOS was associated with lower survival rates of PDAC patients. iNOS/NO signaling can phosphorylate FoxO (Forkhead box-O) and inactivate it through the activation of ERK and PI3K/AKT signaling [46], resulting in impaired functions of cell cycle arrest, DNA repair, and apoptosis [47]. In addition, pancreatic cancer cells from iNOS-deficient mice exhibit diminished migration and invasion, which may be attributed to the lack of NO-induced activation of c-Src and impairment of E-cadherin junctions between cancer cells [46].

As stated earlier, there is a duality of action of NO. Kong G et al., concluded from a study of seventy-two pancreatic cancer tissue specimens that iNOS played a role in causing cancer cell apoptosis [48]. Beyond that, a previous study found the axon guidance factor netrin-1 upregulated NO production to activate protein phosphatase 2A (PP2A) phosphatase and decrease c-Jun phosphorylation. The enhancement of PP2A activity can suppress integrin β4 expression through damaging the MEK/ERK pathway [49]. The impairment of the MEK/ERK/c-Jun pathway inhibits the growth of PDAC cells in vivo, which means NO plays an anti-cancer role in this mechanism.

In addition, the anti-cancer effect of NO is also reflected in the inhibition of proliferation and invasion of pancreatic cancer cells. In a study of investigating the effect of NO on the expression of insulin receptor substrate (IRS)-1 and insulin/insulin-like growth factor (IGF)-I in pancreatic cancer MIAPaCa-2 cells, it was found that NO donor reduced the expression of IRS-1, which is related to the stimulation of growth signaling pathway and cancer proliferation in pancreatic cancer [50,51]. Besides, it suppressed the phosphorylation of Akt/PKB and glycogen synthase kinase-3β (GSK-3β) induced by inhibition of insulin/IGF-I. This was confirmed by a selective iNOS inhibitor 1400W, revealing the function of iNOS/NO in insulin/IGF-I signaling. However, meanwhile, NO also increases phosphorylation of ERK-1/2, leading to cell survival, which seems to contradict its anticancer effects [51].

The outcome of NO biological functions is the result of a combination of multiple factors and multiple pathways. Thus, Sugita H et al., suggested that the inhibitory effect of NO on the proliferation and invasion of cancer cells in their experiment may have been balanced by the phase opposition of the PI3K-Akt and Ras-ERK pathways [51].

### 2.7. Ovarian Cancer

Ovarian cancer is the fifth leading cause of death related to cancers in female and the most fatal type among all female reproductive cancers [52]. Mounting studies show that NO signaling pathway can impact the protein phosphorylation in ovarian cancer, which may play a role in cell apoptosis, proliferation, and migration.

STAT3, one of the key molecules involved in cell proliferation and survival, is constitutively active in tumor cells without tyrosine receptor kinases. It contains the transcriptional regulatory activity and regulates the expression of antiapoptotic genes and proliferation regulatory genes [53]. Evidence obtained from SK-OV-3 and OVCAR-3 ovarian cancer cell lines showed that high level of NO donors, including spermine nitric oxide complex hydrate (SPER/NO) and diethylenetriamine nitric oxide adduct (DETA/NO), induced apoptosis of both ovarian cancer cell lines accompanied with decreasing of STAT3 and Akt protein phosphorylation. Based on the results, this research suggested that NO donor could induce ovarian cancer cell apoptosis through inhibiting STAT3 and Akt phosphorylation, which was confirmed by incubating ovarian cancer cells with selective inhibitors of STAT3 and Akt protein [54]. However, NO also induces anti-apoptosis of ovarian cancer cells through modulating STAT3 phosphorylation. There is a consensus that NO contains pleiotropic biological activity depending on its concentration in the experimental condition. Therefore, the type of NO donor, its half-life, and type of ovarian cancer cell line can impact the role of NO in ovarian cancer cells. S-nitroso-N-acetylpenicillamine (SNAP), an NO donor, can rescue the inhibition of STAT3 phosphorylation induced by a certain antitumor drug to prevent apoptosis of human ovarian cancer OVCAR3 and SKOV3 cells at low concentration [55]. Survivin, highly expressed in tumor cells, acts as a major cytoprotective factor and regulates the apoptosis of ovarian cancer cells [56]. It is reported that NO can impact the expression of survivin through modifying the phosphorylation of upstream proteins to regulate the cell apoptosis in OVCAR3 and SKOV3 human ovarian cancer cell lines, depending on the concentration of NO. High concentration of NO inhibits the expression of survivin by increasing the phosphorylation of p38 MAPK to induce the ovarian cancer cell apoptosis, which is abrogated by pretreatment of p38 MAPK inhibitor [57]. However, low concentration of NO prevents drug-induced apoptosis of ovarian cancer cells through a different signaling pathway. Low amounts of iNOS expressed in ovarian cancer cells stimulate the phosphorylation of PI3K/Akt and subsequently promote the expression of survivin. The results are further confirmed by treating ovarian cancer cells with PI3K/Akt-inhibitor, which reverses the induction of survivin and enhances cell apoptosis [57]. In addition, NO can regulate cell apoptosis through a cGMP-dependent signaling pathway. Research demonstrates that 1H-[1,2,4]oxadiazo-lo[4,3-a]quinoxalin-1-one (ODQ), an inhibitor of sGC, stimulates the phosphorylation of p53 and then induces cell apoptosis in OV2008 human ovarian cancer cell line. Based on this research, it is indicated that the NO signaling pathway may be associated with the regulation of p53 phosphorylation, resulting in cell apoptosis [58].

Except for affecting cell apoptosis, NO signaling pathway also regulates cell proliferation via modulating protein phosphorylation in ovarian cancer. Evidence shows that S-nitrosoglutathione (GSNO), another NO donor releasing NO slowly, inhibits the cell proliferation in chemoresistant ovarian cancer cell lines. GSNO attenuates STAT3 and Akt phosphorylation induced by growth factor and reduces the basal level of their phosphorylation forms [59], suggesting that GSNO may inhibit cell proliferation by regulating the growth factor-induced signaling pathway in ovarian cancer cells. Moreover, Shailendra Giri et al., demonstrated that GSNO may inhibit the phosphorylation of key ovarian cancer-promoting protein through nitrosylation, indicating the potential therapeutic role of nitrosylating agents [59]. Furthermore, sepiapterin, the precursor of a critical cofactor for nitric oxide synthase, can stimulate cell proliferation and migration in SKOV3 human ovarian cancer cell line, accompanied by activation of ERK, Akt and p70S6K. These effects induced by sepiapterin are blocked by NO synthase inhibitor, suggesting that NO may affect sepiapterin-induced cell proliferation through modulating the phosphorylation of proteins in the mitogenic signaling pathway [60].

### 2.8. Neuroblastoma

Accumulating research shows that NO mainly regulates cell apoptosis in neuroblastoma through modulating the phosphorylation of key proteins in several signaling pathways. JNK, as a key factor in cell signaling, regulates a wide range of biological activities, including apoptosis through phosphorylating the downstream protein c-Jun on Ser-63 and Ser-73 [61]. Mounting evidence shows that in neuroblastoma, high concentration of NO can activate JNK and then increase the phosphorylation of c-Jun indirectly, resulting in cell apoptosis. In this process, Lei L et al., indicated that NO-induced activation of JNK may only phosphorylate c-Jun on Ser-63 in human neuroblastoma cell lines [62]. Moreover, NO may also impact the phosphorylation of ERK and p38 MAPK to induce cell apoptosis in neuroblastoma. Evidence obtained from the SH-EP1 human neuroblastoma cell line shows that high concentration of SNP stimulates the phosphorylation of ERK and p38 MAPK, contributing to cell apoptosis. SNP-induced cell apoptosis can be reversed by ERK and p38 MAPK inhibitor, respectively [63]. Tetsuaki N et al., confirmed the hypothesis in a different neuroblastoma cell line, the SH-SY5Y cell line [64]. Combined with the results mentioned before, they found that SNP also induced the increase of intracellular Ca^2+^ concentration significantly and all these effects induced by SNP were abolished after treating the Na^+^/Ca^2+^ exchanger (NCX) inhibitor, suggesting that Ca^2+^ influx via NCX may mediate the SNP-induced protein phosphorylation. In addition, the increase of intracellular Ca^2+^ level induced by SNP can be inhibited by a cGMP-dependent protein kinase (PKG) inhibitor, resulting in the inhibition of SNP-induced cell apoptosis [64]. Taken together, these results indicate that NO may activate NCX via the cGMP/PKG signaling pathway to promote the influx of Ca^2+^, resulting in stimulating cell apoptosis through phosphorylation of ERK and p38 MAPK.

Due to the pleiotropic function of NO, it can also protect neuroblastoma cells against apoptosis through modulating a different signaling pathway. There is a consensus that PI3K/Akt pathway is essential to cell survival [65]. Evidence shows that SNP promotes cell survival in neuroblastoma treated with H_2_O_2_, accompanied by the increase of Akt phosphorylation. These effects induced by SNP can be reversed by the inhibitor of PI3K, suggesting that SNP may modulate the PI3K/Akt signaling pathway to prevent the apoptosis of neuroblastoma cell [66]. Additionally, in SNP-treated neuroblastoma cell line, the phosphorylation of Bad and its interaction with neural cell type protein 14-3-3β increase significantly, suggesting that SNP may induce the interaction between p-Bad and 14-3-3β to block the cell apoptosis cascade. Based on these results, it is predicted that NO/cGMP/PKG may be involved in SNP-induced anti-apoptosis of neuroblastoma to regulate the phosphorylation of Akt and Bad. This hypothesis has been confirmed by treating neuroblastoma cells with sGC and PKG inhibitors respectively, resulting in the inhibition of these effects induced by SNP [66].

## 3. The Therapeutic Effect of Nitric Oxide

Nitric oxide was first discovered by Ferid Murad for its role in promoting cardiovascular dilation. In oncology, NO has been discussed as an oncogene in the past. However, recently, it was discovered that the proper concentration of NO has a role in the cancer cell cycle, death, metastasis, migration, and angiogenesis [67,68]. Therefore, NO and its donor drugs have been increasingly used in cancer chemoprophylaxis, radiotherapy, chemotherapy, and immunotherapy [69]. At low concentration, NO includes in the activation and phosphorylation of ERKs, Akt/mTOR, STAT and Ras signal pathway to enhance angiogenesis and metastasis and stimulate cancer cell progression. However, as an effective vasodilator, NO, is also used in delivering the antitumor drug. In this situation, narrow vessels around the tumor are dilated and blood flow increases, thus the drug can efficiently function [70]. While at higher concentration, NO intends to depress tumor development by inducing cell apoptosis, sensitizing tumors to radiotherapy, reversing resistance to chemotherapy, and declining the angiogenic and metastatic cascades [9]. Also, the study showed that ERK-P, Akt-P, and p53 are correlated with a high concentration of NO, thus inhibiting cancer progression [13].

NO donor commonly found in cancer including organic nitrates, sodium nitroprusside, S-nitrosothiols, sydnonimines, DETA/NO, and JS-K [71]. Organic nitrates, sodium nitroprusside, and S-nitrosothiols have the antitumor function in radiosensitization during therapy. Sydnonimines induce cell injury by DNA damage, increasing protein nitration and decreasing mitochondrial respiration chain. Both organic nitrates and DETA/NO can reduce doxorubicin resistance. A study showed that a low level of DETA/NO can reverse the resistance of human breast cancer to chemotherapeutic agents 5-FU and doxorubicin [72]. Moreover, JS-K can specifically target glutathione (GSH) which is overexpressed in cancer cells [73]. SNOHSA, a NO donor significantly inhibited hypoxia-induced autophagy by inhibiting the phosphorylation of JNK1 and the expression of its downstream molecule Beclin1 in the cancer cell, thereby inhibiting drug resistance [74]. Therefore, the combination of NO donor and gas therapy with other anti-cancer therapies has achieved further tumor suppression including reducing resistance to oncology drugs. It is indicated that NO can reduce the expression of P-glycoprotein and ATP-binding cassette transporters, thus decreasing the multidrug resistance of tumor therapy. NO stimulated nanosystem can promote the accumulation of doxorubicin in blood and reduce its resistance [75,76]. To conclude, Since NO plays an important role in tumor development and inhibition, further researches on its related drug and signal pathways are necessary. Those exogenous deliveries of NO drugs have different inhibitory mechanisms inducing DNA damage, radiosensitization of cancer cell, and reducing multidrug resistance. However, since more NO donor drugs exhibit experiment on the animal instead of the human body, More clinical experiments on the tumor of various organs should be completed in the future.

## 4. Conclusions

This review briefly describes the effect of NO on pathways or protein phosphorylation processes in eight cancers, which cause either positive or negative outcomes.

**Figure 3:**
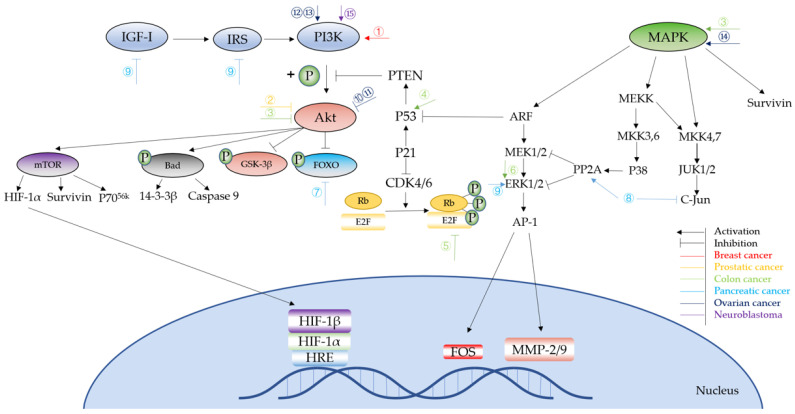
Signaling pathways of NO in different cancer types. ①Pretreatment: NOS2 Result: block apoptosis. ②Pretreatment: NO-sulindac Result: inhibit invasion, increase survival and induce ability to adapt to hypoxia. ③Pretreatment: NO-ASA Result: inhibit cancer growth. ④Pretreatment: NO Result: inhibit cell cycle and apoptosis. ⑤Pretreatment: iNOS-induced NO Result: colitis and anti-apoptosis. ⑥Pretreatment: NO Result: metastasis and invasion. ⑦Pretreatment: iNOS/NO Result: impaired cell cycle arrest, DNA repair and apoptosis. ⑧Pretreatment: Axon guidance factor retrin-1 upregulate NO Result: anti-cancer. ⑨Pretreatment: NO donor Result: inhibit proliferation and invasion. ⑩Pretreatment: GSNO Result: inhibit cell proliferation. ⑪Pretreatment: SPER/NO, DETA/NO Result: apoptosis. ⑫Pretreatment: Sepiapterin Result: cell proliferation. ⑬Pretreatment: iNOS Result: cytoprotection. ⑭Pretreatment: NO Result: apoptosis. ⑮Pretreatment: SNP Result: apoptosis.

Based on the above, it is clear that NO affects cell growth, apoptosis, angiogenesis, cancer invasion, and metastasis mainly through influencing the phosphorylation status of phase transition proteins, the PI3K/Akt pathway, and MAPK pathway, as well as some downstream proteins and transcription factors (Figure 3). Compared to the two-fold effect of NO on anti-cancer effects, which may be related to the NO concentration and its duration of action, NO induced by iNOS is prone to show a more complex regulation of cancer development. iNOS-induced NO have cytotoxic and cytostatic functions on tumor cells [10], but is also involved in cancer development and progression. Perhaps more research is needed in the future to determine the exact role of iNOS in cancer development and whether this role is tumor specific.

Many NO-releasing compounds are produced; however, more in-depth research is still needed. Not only does the drug itself need to be stable and have few side effects, but it also needs to avoid the cancer-promoting effects of NO and extend its anti-cancer benefits as much as possible. For example, NO-sulindac, as described above, can reduce the tolerance of prostate cancer to chemotherapy under hypoxic conditions by inhibiting HIF-1α, which can be used to exploit a better treatment by increasing the sensitivity of hypoxic cells to chemotherapy and radiation therapy [34,77]. In addition, novel NO-releasing biomaterials for tumor-targeted therapy are designed for a better control of NO delivery to overcoming drug resistance in chemotherapy [78].

The introduction of NO and NO synthetic drugs may be a new direction for cancer treatments, or they can be used as an adjunct to reduce therapy resistance, but more research is urgently needed to achieve their clinical application.

## Data Availability

Not applicable.

## Appendix A

**Table A1:** The impact of the NO pathway on partial pathways and proteins in different types of cancer.

Cancer Types	Experimental Cells	Signaling Pathway/Protein	Pre-Treatment	NO/iNOS/eNOS Level	Result	Ref.
Colon cancer	HT-29 Colon Cancer Cell	↑Phosphorylation of JNK, p38 MAP kinases, cJun and ATF-2; Activates AP-1 Complex	NO-aspirin	NO↑	Inhibit cancer cell growth	[14]
Breast cancer	ZR-75-30, BT-474, MCF-12F	Akt and ERK1/2 phosphorylation	Inhibitor of NO synthesis	NOS↑	Tumor cell growth and proliferation	[18]
Breast cancer	4T1 murine breast cancer cells	Akt phosphorylation	4T1 cancer cell inoculation	NOS2↑	Tumor cell growth and proliferation	[19]
		eNOS phosphorylation		NO↓	Tumor metastasis	
Lung cancer		Decreasing activity and phosphorylation of eNOS	4T1 cancer cell inoculation	NO↓	Metastasis	
Breast cancer	MDA-MB-231 breast cancer cells	Akt phosphorylation	TIMP-1 silencing	NO↑	Tumor cell growth and proliferation	[20]
Breast cancer	MDA-MB-468 breast cancer cells	S-nitrosylation of Ras, Ets-1, MAPK	-	NO↑	Tumor metastasis	[21]
Breast cancer	The murine cell line 66CL4, human breast cancer cell line MCF-7, human triple negative breast cancer (TNBC) cell lines	VEGF, phosphorylation of γ-H2AX foci and RAD51 foci	Glucocorticoids	NO↑	Angiogenesis	[23]
Breast cancer	Human breast cancer cell lines MDA-MB-468, ZR 75-30 and MDA-MB-231	Dephosphorylation of ERK and Akt	Sodium orthovanadate and antisense oligonucleotides	NO↑	Cancer cell growth	[24]
Breast cancer	Human breast cancer cell lines MDA-MB-231, MCF-7, and MDA-MB-468	Raf/MEK/ERK and PI-3 kinase/Akt pathways	diethylenetriamine-NONOate (DETA-NONOate)	NO↑	Proliferation of cancer cell	[25]
Lung cancer	C10 and E10 cells, LM1 and LM2 cells	ERK1/2 kinase phosphorylation	ERK 1/2 kinase inhibitor U0126	NO↑	Tumor proliferation and growth	[27]
Lung cancer	Human colon carcinoma cell line and human lung adenocarcinoma cell line	Tyrosine phosphorylation and p53 gene accumulation	Retroviral vector DFG-iNOS	NO↑NOS↑	Metastasis	[28]
Lung cancer	Bronchiolar Clara cells	Vascular endothelial growth factor	iNOS -	NO↑iNOS↑	Angiogenesis	[29]
Lung cancer	NCI-H157 and NCI-H522	Tyrosine phosphorylation and MMP expression	-	NO↑NOS↑	Angiogenesis	[30]
Lung cancer	Human non-small lung cancer (NSCLC)-derived cell line H460, H23, and H292	Akt phosphorylation	-	NO↑	Promote cancer stem cell	[31]
Lung cancer	NCI-H460 cells	Inhibition the ubiquitination of Bcl-2 protein	Sodium nitroprusside, dipropylenetriamine NONOate and cisplatin	NO↑	increase cell death resistance	[33]
Ovarian cancer	SKOV-3 and OVCAR-3 cell lines	↓Phosphorylation of STAT3, Akt	Spermine nitric oxide complex hydrate (SPER/NO), diethylenetriamine nitric oxide adduct (DETA/NO)	NO↑	Cancer cell apoptosis	[54]
Ovarian cancer	SKOV-3 and OVCAR-3 cell lines	↑Phosphorylation of STAT3	Arctigenin, S-nitroso-N-acetylpenicillamine (SNAP)	NO↑	Inhibit arctigenin- induced cell apoptosis	[55]
Ovarian caner	SKOV-3 and OVCAR-3 cell lines	↑Phosphorylation of p38MAPK, ↓expression of survivin	High concentration of NO donors (SNP, SNAP) or overexpression of iNOS	NO↑	Cancer cell apoptosis	[57]
	-	↑Activation of PI3K/Akt/survivin signaling pathway	Low concentration of NO donors (SNP, SNAP) or ectopic expression of low amounts of iNOS	NO↑	Cytoprotection	
Ovarian cancer	SKOV-3,OVCARs, A278, C200, OVCAR4, PE01 and PE04 cell lines	↓Phosphorylation of Akt, STAT3 and p42/44; ↑Nitrosylation of EFGR, Akt, STAT3 and NFκBp65	S-nitrosoglutathione (GSNO)	NO↑	Inhibition of cancer cell growth	[59]
Ovarian cancer	SKOV-3 cell line	↑Phosphorylation of ERK, PI3K/Akt and mTOR/p70^S6K^ signaling pathway	Sepiapterin	NO↑	Increased cancer cell growth and migration	[60]
Neuroblastoma	SH-Sy5y and SHEP cell lines	↑Phosphorylation of JNK	SNP	NO↑	Cancer cell apoptosis	[62]
Neuroblastoma	SH-EP1and SH-SY5Y cell lines	↑Phosphrylation of ERK1/2 and p38 MAPK	SNP	NO↑	Cancer cell apoptosis	[63,64]
Neuroblastoma	SK-N-MC cells	↑Activation of PKG/PI3K/Akt signaling pathway, ↑Phosphorylation of Bad	SNAP	NO↑	Inhibit H_2_O_2_- induced cell apoptosis	[66]
Gastric cancer	BGC-823 cell line	↓Phosphorylation of Akt	SNP	NO↑	Inhibition of cancer cell growth	[44]
Gastric cancer	AGS cell line	↓Phosphorylation of EGFR and ERK1/2	SNP, L-arginine and overexpression of PKG II	NO↑	Inhibition of cancer cell growth	[45]
Colon adenocarcinoma; Pancreatic adenocarcinoma; Skin carcinoma; cervix adenocarcinoma; Breast adenocarcinoma	SW480, HT-29, HCT-15, and LoVo human colon adenocarcinoma cells; BxPC-3 human pancreatic adenocarcinoma cell; A431 human skin carcinoma cell; HeLa human cervix adenocarcinoma cell; MCF-7 human breast adenocarcinoma cell	↑Cyclin B1, phosphorylation of Cdk1, oxidative stress, ROS; ↓cyclin D1 and Cdc25C	NO-aspirin	NO↑	Inhibit cancer cell growth	[37]
Colon cancer	Human colon cancer cell lines RKO and RKO-E6; RKO_β-cat_ cell	↓NO–induced release of cytochrome c, p53; ↑NO–induced suppression of the antiapoptotic protein, Bcl-xL and Akt phosphorylation	Overexpression β-catenin; DETA- NONOate; sodium nitroprusside	-	Blocks NO–induced cancer cell apoptosis	[39]
Colorectal adenocarcinoma	SNU-1040 cell line and HCT-116 cell line	↑Phosphorylation of p53	AdiNOS; SNAP	iNOS↑, NO↑	Cancer cell apoptosis; Inhibit cancer cell growth	[40]
Colon cancer	HCT116, HT29, DLD-1, and HCT15 colon cancer cell line; iNOS^+/+^ and iNOS^−/−^ mice	Rb hyper-phosphorylation and inactivation; ↑activation of sGC/cGMP/PKG pathway, PI3K/AKT and MEK/ERK1/2 pathways	Spermine NONOate	NO↑	Increase cancer cell growth; reduce cancer cell apoptosis	[41]
Colon adenocarcinoma	WiDr, SW 480, SW 620 colon adenocarcinoma cells	↑Phosphorylation of ERK1/2, activation of AP-1, nuclear translocation of Fra-1/Fra-2, MMP-2/9, RhoB, Rac-1, β-catenin and cyclin D1, cGMP; ↓caspase-8, caspase-3/9 activation and PARP cleavage	SNAP	NO↑	Increase cancer proliferation, metastasis and invasion; reduce cancer cell apoptosis	[43]
Pancreatic ductal adenocarcinoma	LSL-Kras^G12D^; LSL-Trp53^R172H/+^; Pdx-1-Cre (KPC) pancreatic cancer mouse model and NOS2-deficient KPC (NKPC) mice; primary pancreatic tumor tissue	↓Phosphorylation of FOXO, activation of ERK and PI3K/AKT signaling pathway;	iNOS deficiency	iNOS↓ NO↓	Decrease cancer cell growth; cancer cell apoptosis	[46]
Pancreatic cancer	Seventy-two pancreatic adenocarcinoma tissue specimens	↑iNOS-induced NO	-	-	Cancer cell apoptosis	[48]
		↑COX-2 activate NF-κB			Reduce cancer cell apoptosis	
Pancreatic ductal adenocarcinoma	MiaPaCa II, AsPC-1, and 293T cells; four-week-old SCID-beige mice; Balb/c NU\NU mice	↑NO, PP2A; ↓MEK/ERK pathway, recruitment of phosphorylated c-Jun, integrin β4 expression, ITGB4	Netrin-1	NO↑	Cancer cell apoptosis; inhibit cancer cell growth	[49]
Pancreatic Cancer	MIAPaCa-2, Panc-1, MCF-7, MB 468, and HepG2 cells	↓Phosphorylation of insulin receptor/IGF-I receptor, IRS-1, IGF-I receptor, Akt/PKB, GSK-3β; ↑phosphorylation of ERK-1/2	SNAP, GSNO, 1400W	NO↑	Inhibit cancer proliferation and invasion	[51]
BPH, low- and high-grade PIN and prostatic carcinoma	Tissue samples of BPH, low-grade PIN, high-grade PIN and primary prostatic adenocarcinomas	↑iNOS, iNOS-induced NO	-	NO↑ iNOS↑	Induce cancer cell metastasis and tumorigenesis	[36]
Prostate cancer	PC-3 prostate cancer cell line	↓Activity of HRE promoter, Akt phosphorylation; inhibit HIF-1α translation	NO-sulindac	NO↑	Inhibit cancer cell growth and invasion; Cancer cell apoptosis	[34]
